# CD164 promotes tumor progression and predicts the poor prognosis of bladder cancer

**DOI:** 10.1002/cam4.1607

**Published:** 2018-07-18

**Authors:** Xiao‐Guang Zhang, Tong Zhang, Chang‐Ying Li, Ming‐Hao Zhang, Fang‐Min Chen

**Affiliations:** ^1^ Department of Urology Third Central Hospital of Tianjin Tianjin China; ^2^ Department of Urology Provincial Hospital Affiliated to Shandong University Jinan China; ^3^ Tianjin Institute of Urology Tianjin China

**Keywords:** bladder cancer, CD164, CXCR4, prognosis, progression

## Abstract

CD164 was found to play a role in many malignant diseases. But the roles of CD164 in human bladder cancer have not yet been studied. The object of our study was to investigate the functions of CD164 in urothelial bladder carcinoma. The immunohistochemistry (IHC) was performed to evaluate the associations between the expression level of CD164 and clinical‐pathological features of patients, and IHC was used to analyze the relationship between CD164 and CXCR4 in tumor tissues. Real‐time qPCR and Western blot were used to measure the expression of relevant genes. The roles of CD164 in tumor cells and tissues were investigated by in vitro and in vivo experiments. The results of immunohistochemistry found that CD164 was associated with clinical and pathological features of patients. High level of CD164 was related to the distant metastasis and vascular invasion of bladder cancer patients. In vitro, by silencing of CD164, the proliferation, migration, and invasion of tumor cells were inhibited significantly by regulating related proteins such as Ki67, proliferating cell nuclear antigen, matrix metalloproteinases‐2, and matrix metalloproteinases‐9. In vivo, knocking‐down of CD164 could reduce the growth and metastasis of tumors in mice. In addition, a co‐expression was found between CD164 and CXCR4 in tumor tissues. In conclusion, our study demonstrated that CD164 was associated with the poor clinical outcomes of BC patients. Silencing of CD164 could inhibit the progression of tumors in vivo and in vitro, which may become an effective target in the treatment of bladder cancer.

## 1 INTRODUCTION

Bladder cancer (BC) is the ninth most common cancer and the thirteenth deadliest worldwide with 81190 newly diagnosed cases and 17240 deaths estimated in USA, 2018.^1^ Most of bladder cancers are derived from urothelial cells, and roughly 75% of patients are nonmuscle invasive bladder cancer (NMIBC) and 25% have muscle invasive (MIBC) or metastatic disease.^2^ Despite the continuous development of medical techniques, the diagnostics, treatments, and survivals for bladder cancer have been largely unchanged since the 1990s.^3^ Although NMIBC patients with a better prognosis than MIBC, and even through tumors can be successfully identified and removed before they become invasive, BC has a high rate of recurrence and progression.^4^ Cystoscopies are the gold standard for diagnosis of BC, but they are expensive and uncomfortable.^5^ And they sometimes fail to find types of tumor that have not yet become invasive but are aggressive, such as carcinoma in situ.^6^ With the limitations of present diagnosis and treatment, researching into cancer genomics, risk markers, and targeted therapies could hold the key to progress against this malignant disease.

CD164, also known as endolyn or MGC‐24v, is a member of sialomucin family, which is conversed and encoded by CD164 gene located on human chromosome 6q21.^7^, ^8^ CD164 was first identified in primitive CD34^+^ hemopoietic progenitor cells and bone marrow stromal cells and has been found to be involved in proliferation, migration, and adhesion of these cells.^9^, ^10^, ^11^ By facilitating the adhesion and migration of human CD34^+^ cells to bone marrow stroma, CD164 was proposed to regulate hematopoiesis.^12^ In human cancers, CD164 was reported played roles in many different cancers. For instance, CD164 has been reported for the maintenance and progression of human tumors, such as human glioma,^13^ lung cancer,^14^ ovarian cancer,^15^ and prostate cancer.^16^ Besides, some microRNAs such as miR‐124 and miR‐219 were found that could suppress the proliferation, migration, and invasion of tumor cells by targeting CD164.^17^, ^18^ However, the roles of CD164 in BC have not yet been studied and are still unclear.

In this study, we systematically researched the roles of CD164 in BC. The immunohistochemistry was used to investigate the associations between CD164 and clinical outcomes of BC patients. Then, the functions of CD164 in tumor proliferation, migration, and invasion were studied in vivo and in vitro. According to these, we hoped to find an effective marker which may become the potential therapeutic target of BC.

## 2 MATERIALS AND METHODS

### 2.1 Patients and samples

One hundred and fifteen patients clinically and pathologically diagnosed with urothelial carcinoma of bladder in third central hospital of Tianjin and provincial hospital affiliated to Shandong university were included in our study. Tumor tissues were obtained after the first surgical treatment (TURBt or Cystectomy). The clinical and pathological characters such as ages, genders, pTMN stage, tumor grade, tumor sizes, lymph metastasis, and vascular invasion were record. The tumor stage was classified by 2009 UICC TMN staging and tumor grade was determined according to the 2004 WHO/ISUP classification.^19^, ^20^ Written informed consents and approval from the institution ethics commission of third central hospital of Tianjin and provincial hospital affiliated to Shandong university were obtained.

### 2.2 Immunohistochemistry and scoring

Bladder tumor specimens were fixed by 10% v/v formalin solution, and embedded in paraffin. One hundred and fifteen paraffin‐embedded tissues collected from the third central hospital of Tianjin were sliced into 4‐μm sections and baked at 65°C for 30 minutes. Then, the sections were performed with EDTA (pH = 8.0) and 3% H_2_O_2_ in methanol. The tissues sections were cultured with: anti‐CD164 antibodies (rabbit; 1:200; sigma), anti‐CXCR4 antibodies (rabbit; 1:200; sigma) overnight at 4°C in a moist chamber. Then, the second antibody was added and incubated at room temperature for 1 hour. The sections were counterstained using hematoxylin and incubated with streptavidin‐horseradish peroxidase complex. For the results, CD164 cytoplasmic staining was scored by using 4‐point scales (0, no staining; 1+, light staining at high magnification; 2+, intermediate staining; 3+, dark staining of linear membrane at low magnification). Besides, an immunostaining score (*H*‐score) was measured by the multiplication of CD164 and CXCR4 stained cells and the corresponding intensity score.^21^ According to the distribution of *H*‐scores, the CD164 and CXCR4 were divided into high and low expression groups, respectively.

### 2.3 Cell culture

Human urothelial bladder cancer T24 and 5637 cell lines were purchased from the Cell Bank of the Chinese Academy of Sciences (Shanghai, China). According to the instructions, both of T24 and 5637 cells were cultured in RPMI‐1640 medium containing 1% penicillin streptomycin and 10% fetal bovine serum with 5% CO_2_ at 37°C.

### 2.4 Plasmid construction and Lentiviral transfection

CD164 short hairpin RNA oligonucleotide sequence (shRNA) was used to knock down the expression of CD164 gene. The sequence of CD164 shRNA was as follows: 5′‐TGAGAAAGC TCTCCACTCTGTTCAAGAGACAGAGTGGAGAGCTTTCTCTTTTTTC‐3′. And a scramble shRNA was performed as the negative control. The efficacy of knocking‐down was verified by RT‐qPCR and Western blot. Then, qualified cells were selected for using in later experiments.

### 2.5 RT‐PCR

Total RNA was extracted from tumor tissues and cells using TRIzol reagent (Thermo, American) according to the instructions. RNA was reversely transcripted to cDNA by the cDNA Reverse Transcription Kit (Sangon Biotech, China). Quantitative PCR was performed on a Smart Cycler using SGExcel FastSYBR Mixture (With Low ROX) Plus (Sangon biotech). A comparative threshold cycle (Ct) method, which compares differences in CT values between common control and target RNA, was used to process the real‐time PCR data.^22^ The primer sequences of CD164 were as follows: (Forward) 5′‐TGAGCCCTGAACACCAGAGAG‐3′, and (Reverse) 5′‐AAAGCCAGATGAGCGCTTCTA‐3′.

### 2.6 Western blot

Cells and tissues were lysed in RIPA lysis buffer, and proteins concentrations were measured by using the bicinchoninic acid (BCA) method. Cell/tissue lysates were separated by SDS‐PAGE gels and transferred to PVDF membranes (Thermo, American). Then, the blots were blocked by 5% dry milk and incubated with the primary antibodies: anti‐CD164 (rabbit; 1: 1000 dilution; Cambridge, UK), anti‐β‐actin (mouse; 1:1000 dilution; Cambridge, UK), anti‐Ki67 (rabbit; 1:1000; Cambridge, UK), anti‐PCNA (mouse; 1:500 dilution; Cambridge, UK), anti‐MMP2 (mouse; 1:1000 dilution; Cambridge, UK), and anti‐MMP9 (mouse; 1:1000 dilution; Cambridge, UK) monoclonal antibodies overnight at 4°C. Then, the samples were incubated with the secondary antibody (polyclonal goat anti‐rabbit/mouse, 1:10 000 dilutions, Rockland Immunochemicals Inc, PA) for 1 hour at room temperature and detected by chemiluminescence.

### 2.7 Colony formation array

Four hundred cells per well of T24 and 5637 cells were seeded into 60 mm dishes and cultured at 37°C in 5% CO_2_. After 10 days, cells were fixated with 10% formaldehyde for 5 minutes and stained 10‐30 minutes with Giemsa. Finally, the colonies were counted using an optional microscope and the colonies with diameters >2 mm were counted. The arrays were performed in triplicate.

### 2.8 MTT array

T24 and 5637 cells were seeded into 24‐well plates with a density of 2 × 10^3^ cells/well and 10 μL of 5 mg/mL MTT reagent was added into each well for 4 hours at 37°C. Then, the media were removed, and dimethyl sulfoxide (DMSO, Sigma) was added. The absorbance was measured at 570 nm. The arrays were repeated in triplicate.

### 2.9 Wound and healing assay

2 ×  10^4^/well of T24 and 5637 cells were seeded in 12‐well plates and grown to a confluence. Then a scratch was created using a sterile 200 μL pipette tip and cells were calculated at 37°C in 5% CO_2_. The distance between the edges of both sides was measured, which represented the ability of cell migration. Three repeated arrays were carried out.

### 2.10 Transwell array

The transwell membrane with Matrigel Substrate (BD, USA) was used to investigate the invasion of tumor cells. 2 × 10^5^ cells were added into the upper chambers, and the low chambers were filled with the RPMI‐1640 supplemented with 10% FBS. The cells were calculated for 24 hours at 37°C in 5% CO_2_. The cells under the surface of the lower chamber were fixed and stained with 0.1% crystal violet. The number of cells was observed from 5 randomly selected photographs under a microscope (magnification, ×200). The arrays were performed in triplicate.

### 2.11 Animal study

All animal experiments in our study were approved by the Animal Care and Use Committee of the third central hospital of Tianjin. BALB/c nude mice (5‐weeks old) were purchased from Slac Laboratory Animal Co. Ltd. (Shanghai, China). 1 × 10^7^ T24 cells transfected with CD164 shRNA and controls were subcutaneously injected into the right armpits of mice. The growth of tumors was monitored over a 7‐week period, and the tumor volumes were measured. After 49 days, tumors were harvested and mice were euthanized. Besides, to investigated the association between CD164 and metastasis of bladder tumor cells. T24 cells transfected with CD164 shRNA and controls were injected into mice tail vein. The metastatic tumors of lung were detected, after 4 weeks, the mice were euthanized, and tumors were harvested.

### 2.12 Statistic analysis

The data in this study were analyzed by SPSS.22.0. Quantitative dates were assessed by mean ± SD. According to the variance homogeneity or not, parametric test and nonparametric test were used respectively. Chi‐square tests were used to analyze the associations between the expression of CD164 and the clinicopathological features. Chi‐square tests and correlation analysis (Pearson and Spearman) were performed to analyze the associations between CD164 and CXCR4. Kaplan‐Meier method was performed to estimate the prognosis of patients. For the results, *P *< .05 for the difference was considered to be significant.

## 3 RESULTS

### 3.1 Association between CD164 and clinical‐pathological features of patients with BC

**Figure 1:**
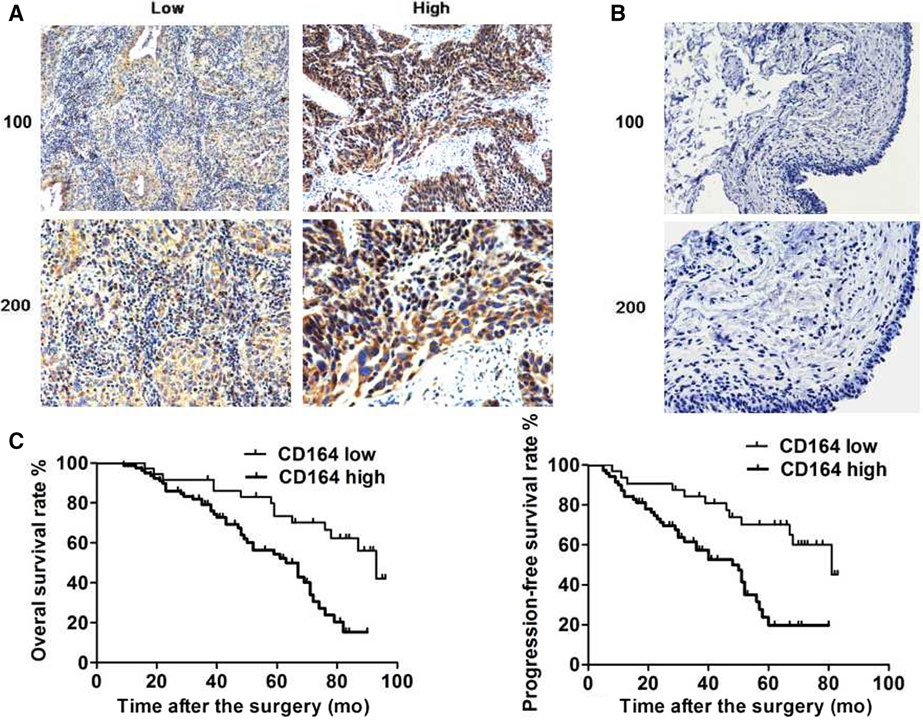
Immunohistochemistry of the expression level of CD164 in bladder tumor tissues and association between CD164 and survivals of patients. A, The typical strong and weak staining of CD164 in tumor tissues by immunohistochemistry. B and C, The associations between CD164 and prognosis of patients were performed by Kaplan‐Meier method, high level of CD164 was associated with short OS and RFS (*P* < .05)

Immunohistochemistry of tumor tissues from 115 patients diagnosed with BC in third central hospital of Tianjin and provincial hospital affiliated to Shandong university was performed to investigated the association between the expression of CD164 and clinical‐pathological characters of patients. Both of the nucleuses and cytoplasm (mainly) of tumor cells were dyed and the typical strong and weak staining of CD164 was shown, while CD164 was negative in the normal bladder tissues (Figure 1A,B). The results showed that CD164 was significantly associated with distant metastasis and vascular invasion (*P *< .05). A high level of CD164 was related to the distant metastasis and vascular invasion of BC. However, no associations were found between CD164 and ages, genders, tumor grade, pTMN stage, and lymph node metastasis (Table 1).

**Table 1:** Relationships of CD164 and clinicopathological characteristics in 115 patients with BC

Feature	All n = 115	CD164 expression		χ^2^	*P*
		Low n = 36	High n = 79		
Age (year)				2.184	.139
<65	46	18	28		
≥65	69	18	51		
Gender				0.332	.565
Male	99	30	69		
Female	16	6	10		
Tumor stage				0.208	.648
T2	35	12	23		
T3/T4	80	24	56		
Tumor grade				1.408	.235
Low	33	13	20		
High	82	23	59		
Lymph node metastasis				1.669	.196
Yes	35	8	27		
No	80	28	52		
Recurrence				1.948	.163
Yes	59	15	44		
No	56	21	35		
Distant metastasis				5.830	.016
Yes	51	10	41		
No	64	26	38		
Vascular invasion				5.967	.015
Yes	64	14	50		
No	51	22	29		

### 3.2 Association between CD164 and prognosis of BC patients

Kaplan‐Meier method was performed to estimate the association between CD164 and the prognosis of patients. Overall survival (OS) and relapse‐free survivals (RFS) were followed up by cystoscopy in our center with every 3 months during the first year, then every 6 months during the following years. The results found that CD164 was significantly associated with the survivals of patients (Figure 1C). As was shown, high level of CD164 was suggested to be related to the short PFS and OS (*P *< .05).

### 3.3 Stably knocking down CD164 in both T24 and 5637 cell lines

**Figure 2:**
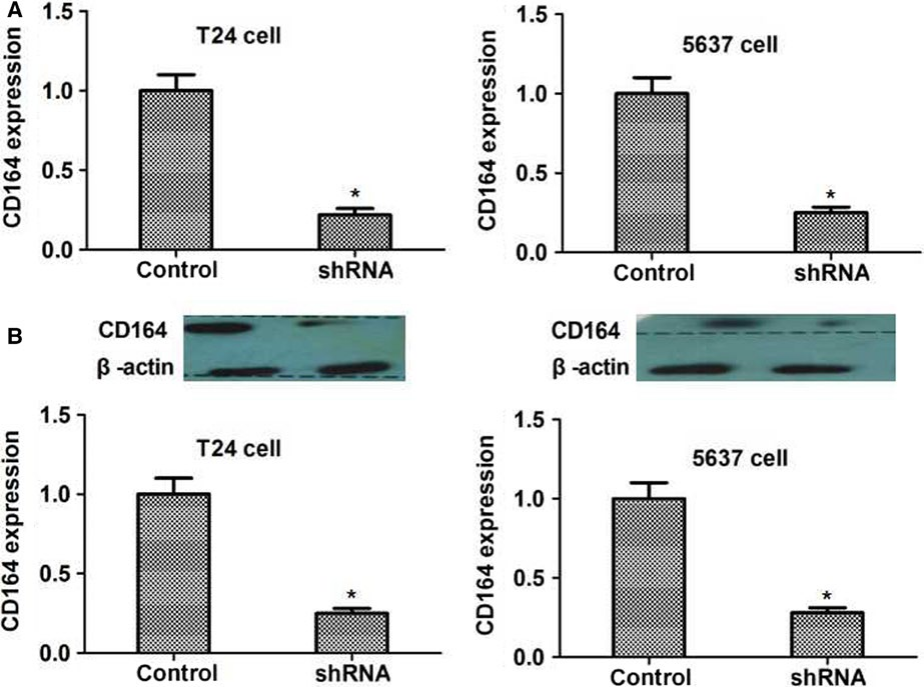
Stably knocking down CD164 by shRNA in both cell lines. The expression of CD164 in T24 and 5637 cell lines transfected by shRNA lentivirus was detected (A) in RT‐qPCR and (B) in Western blot. The expression of CD164 in shRNA group was significantly decreased compared to controls (*P* < .05)

CD164 shRNA was used to knock down the expression of CD164 gene in both T24 and 5637 cell lines. The expression of CD164 in shRNA and control groups was detected in RT‐qPCR and Western blot. The results found that the expression of CD164 was dramatically decreased in shRNA group compared to controls (*P *< .05), which indicated that we have effectively knocked down the expression of CD164 in both cell lines (Figure 2).

### 3.4 Silencing of CD164 inhibited the proliferation of tumor cells by regulating Ki67 and PCNA

**Figure 3:**
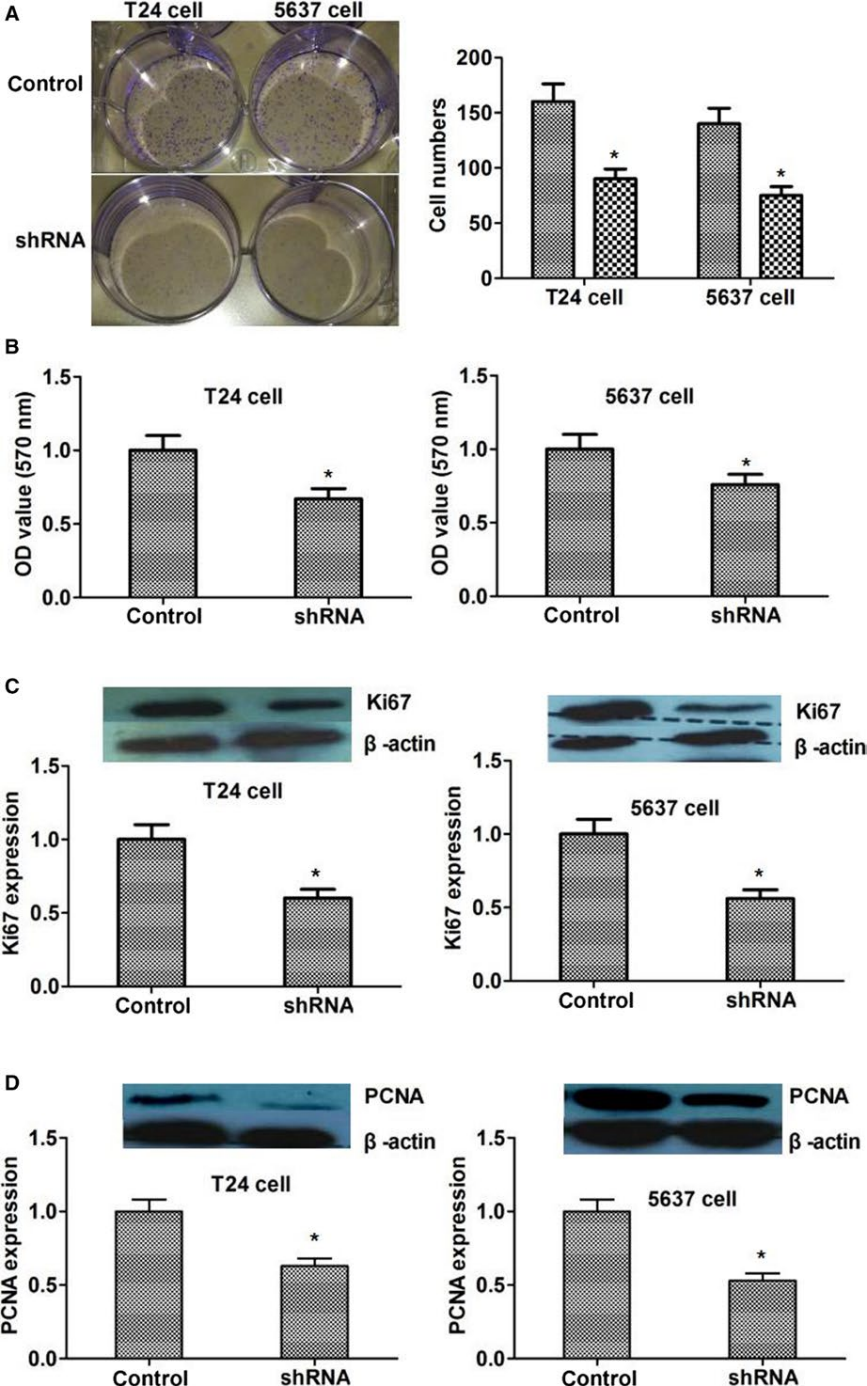
Silencing of CD164 inhibited the proliferation of tumor cells by regulating Ki67 and PCNA. A, The results of colony formation array found that colonies in shRNA group were less than controls (*P* < .05). B, MTT array showed that OD values in shRNA group were decreased (*P* < .05). C and D, The expression of proliferation related proteins Ki167 and PCNA was down‐regulated by silencing of CD164 (*P *< .05)

Colony formation and MTT arrays were performed to investigate the impact of CD164 on the proliferation of tumor cells. The results exhibited that comparing to controls, silencing of CD164 could inhibit the proliferation of both T24 and 5637 cells (*P *< .05; Figure 3A,B). Then, to further explore the mechanism of CD164 in tumor proliferation, some proliferation‐related proteins were identified and detected. The result found that the expressions of Ki67 and proliferating cell nuclear antigen (PCNA) were decreased when we silenced the expression of CD164 (*P *< .05; Figure 3C,D). In conclusion, the results revealed that silencing of CD164 could inhibit the proliferation of tumor cells by regulating the expression of proliferation‐related proteins such as Ki67 and PCNA.

### 3.5 Silencing of CD164 inhibited the migration and invasion of tumor cells by regulating MMP2 and MMP9

**Figure 4:**
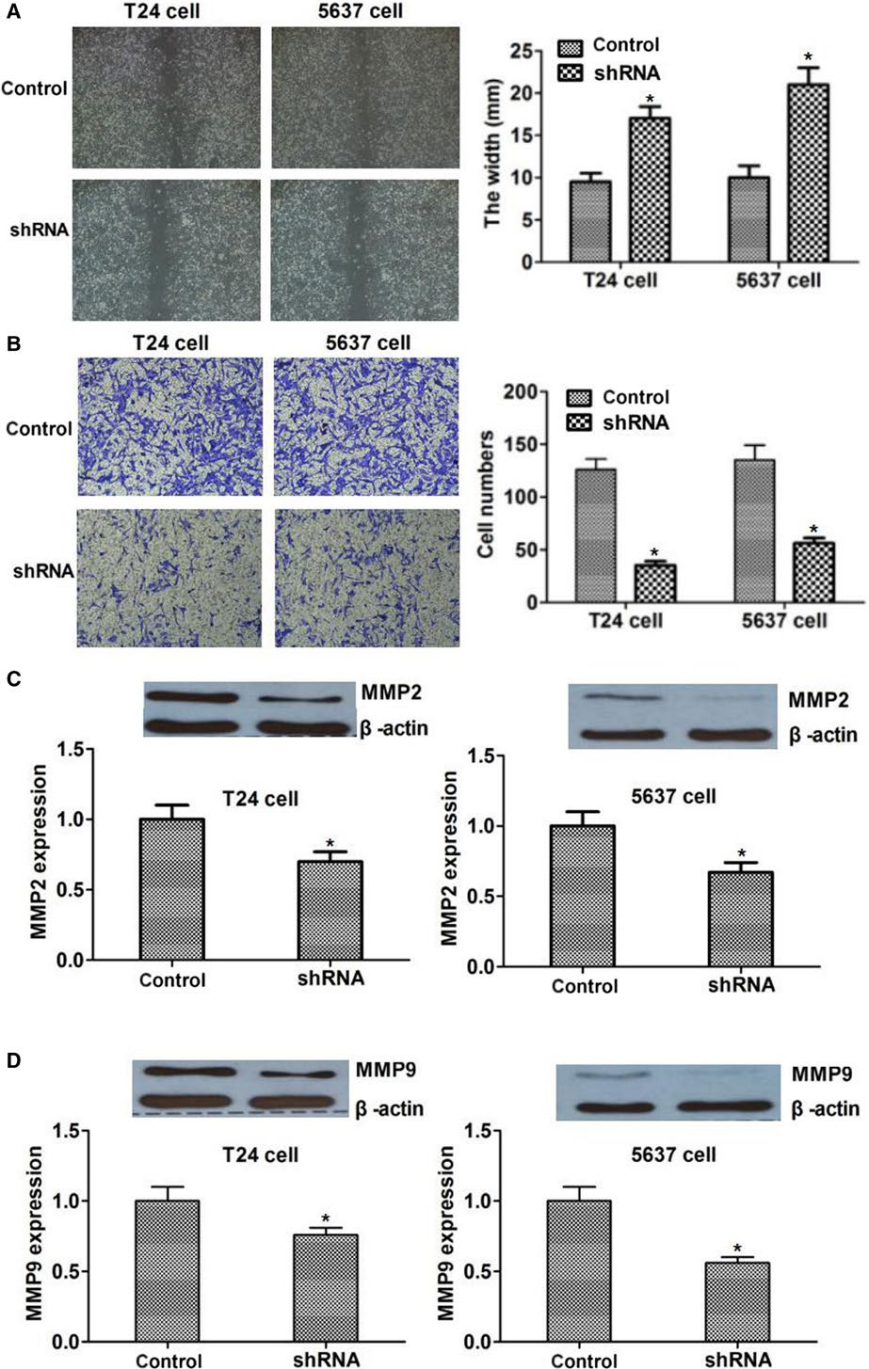
Silencing of CD164 inhibited the migration and invasion of tumor cells by regulating MMP2 and MMP9. A, Wound and healing assay was used to investigate the influence of CD164 on tumor migration. The distance between edges in shRNA group were wider than controls (*P* < .05). B, Transwell array showed that the ability of invasion in shRNA group was decreased significantly (*P* < .05). C and D, The expression of migration and invasion‐related proteins MMP2 and MMP9 was reduced in shRNA group (*P* < .05)

Wound and healing assay and transwell array were used to investigate the associations of CD164 with migration and invasion of tumor cells. The data showed that by knocking down CD164, the abilities of migration and invasion of tumor cells were decreased (*P *< .05; Figure 4A,B). Regarding the mechanism, we examined the expression of proteins that reflected the invasion and migration of tumor cells. By silencing of CD164, the expressions of matrix metalloproteinases‐2 (MMP2) and matrix metalloproteinases‐9 (MMP9) protein were inhibited in our study (*P *< .05; Figure 4C,D). Taken together, we suspected that CD164 could promote the migration and invasion of bladder tumor cells through regulating relevant proteins including MMP2 and MMP9.

### 3.6 Knocking down CD164 restrict the growth and metastasis of tumors in mice

**Figure 5:**
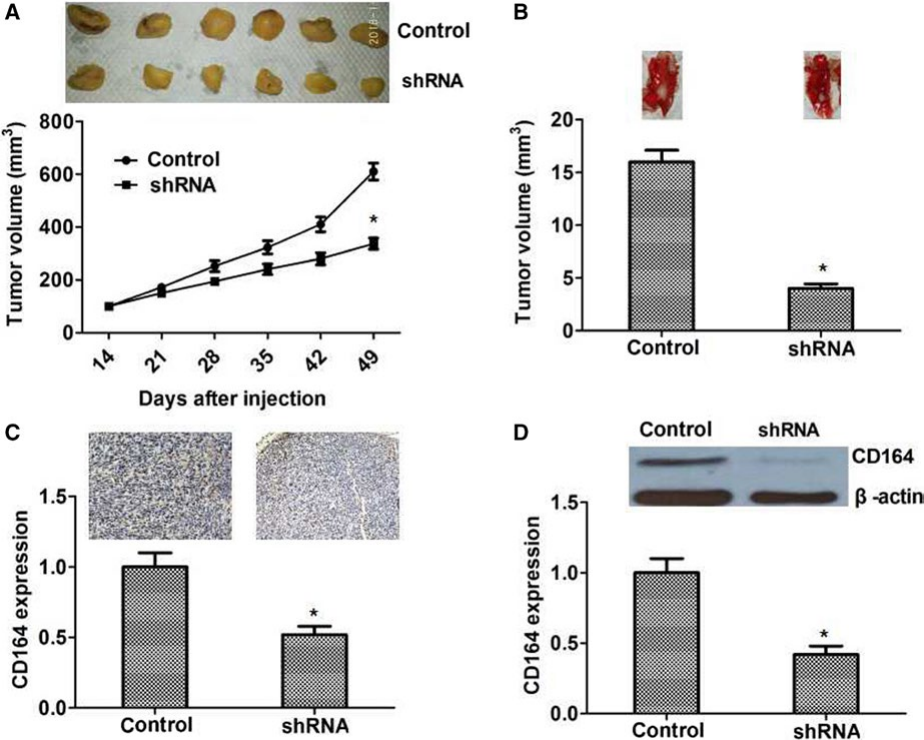
The influence of CD164 on tumor growth and lung metastasis of BC in mice. A, From growth curve, tumors in shRNA group grew more slowly than controls and tumor volumes were smaller in shRNA group (*P* < .05). B, To investigate the influence of CD164 on tumor metastasis of mice, the T24 cells were injected into mice tail vein. The pulmonary metastatic tumors in shRNA group were smaller than controls (*P* < .05). C and D, The expression of CD164 in mice tumors was dramatically decreased by immunohistochemistry and Western blot (*P* < .05), which suggested a successful construction of CD164 knocked down model in mice

To further demonstrate the roles of CD164 in tumor progression, we researched the influence of CD164 on tumor growth and metastasis in mice. Transfected T24 cells in CD164 shRNA and control groups were injected subcutaneously into the right armpits of mice separately. After 2 weeks, tumor volumes were measured every 7 days. From the growth curve, tumors in CD164 shRNA group were grow slower than controls. After 49 days, tumors were harvested and measured. The results demonstrated that by knocking down CD164, the growth of tumors were restricted obviously in mice (*P *< .05; Figure 5A). Besides, T24 cells in 2 groups were injected into mice tail vein to observe the difference of lung metastasis. The results suggested that the metastatic tumors in CD164 shRNA group were obviously smaller than controls (*P *< .05; Figure 5B). Western blot and immunohistochemistry were preformed to detect the expression of CD164 in mice tumors. We found CD164 was dramatically decreased in CD164 shRNA group (Figure 5C,D), which confirmed that an effective and stable knocking‐down of CD164 was built in mice tumors. In summary, our findings demonstrated that silencing of CD164 could restrict the growth and metastasis of tumors in mice.

### 3.7 Co‐expression existed between CD164 and CXCR4 in bladder cancer tissues

**Figure 6:**
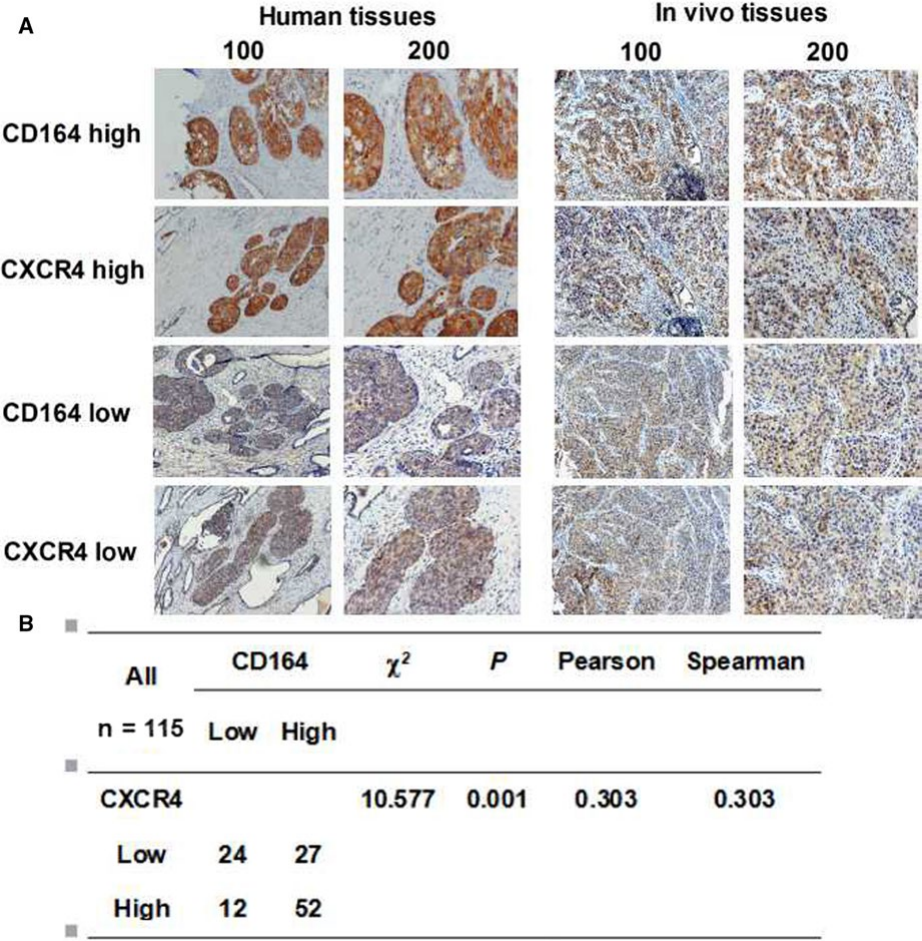
Co‐expression between CD164 and CXCR4 in tumor tissues by immunohistochemistry. A, The immunohistochemistry of CD164 and CXCR4 in 115 samples were performed. The typical strong and weak staining was shown. B, Chi‐square tests, and correlation analysis (Pearson and Spearman) were performed to analyze the associations between CD164 and CXCR4. The results found that an obvious positive correlation was existed (*P* < .001)

As CD164 acted as a CXCR4‐associated sialomucin, the relationship between CD164 and CXCR4 has been researched in many different cancers.^15^, ^23^ In our study, we used the immunohistochemistry to observe the association between the expression of CD164 and CXCR4 in tumor tissues by uninterrupted slicing. The typical staining was shown (Figure 6A). The results found that an obvious positive correlation was existed (Figure 6B). Therefore, we speculated that CD164 possibly played roles in bladder cancer through regulating the expression of CXCR4 and relevant pathways.

## 4 DISCUSSION

As far as we know, there are no reports mentioned about the roles of CD164 in BC. Our study is the first experiment which systematically researched the associations between CD164 and progression of BC. As we know, BC is a common human carcinoma, although primary tumors can be successfully removed, the tumors recur easily and may progress to muscle‐invasive ones.^24^ The traditional standard therapies could restrict the growth and development of tumor. However, they could hardly prevent patients from recurrence and drug resistance.^25^ Therefore, researching on novel potential risk markers may provide new directions and progress for the treatment of BC.

CD164, a member of the sialomucin family, was a mucin that contained sialic acid.^26^, ^27^ As a multifunctional protein, CD164 acted as a surface marker of hematopoietic stem cells, a CXCR4 promoter activity‐enhancing transcription factor, and a stem cell‐specific marker inducer.^9^, ^10^, ^11^ High expression of CD164 was found in several malignant diseases and was associated with clinical outcomes of patients.^13^, ^23^, ^28^, ^29^, ^30^, ^31^ In lung cancer, the positive associations were significantly existed between CD164 expression and tumor size, tumor cell grading, and lymph node involvement.^14^ Besides, a high abundance of CD164 protein was significantly correlated with high‐grade of ovarian tumors.^15^ In our study, the results of immunohistochemistry from 115 patients showed that high expression of CD164 was associated with clinical‐pathological characters such as distant metastasis and vascular invasion. And high level of CG164 was related to the short OS and RFS of BC patients. All these results implied that CD164 may be a risk marker, which played a role in tumor progression.

Previous studies have revealed that CD164 was involved in tumor progression via the regulation of cell proliferation and apoptosis in several cancers.^13^, ^14^, ^15^, ^23^, ^32^ In addition, CD164 was implicated in regulating the migration and invasion of lung cancer and medulloblastoma cells.^17^, ^18^ In vivo, knocking down CD164 could significantly inhibit the tumor growth and metastasis of colon and ovarian cancer in nude mice.^23^ Similarly, in our study, silencing of CD164 could significantly inhibit the proliferation, migration, and invasion of tumor cells in vitro. By knocking down CD164, the growth of tumors subcutaneously injected into mice was dramatically restricted. Besides, the lung metastasis of mice in CD164 shRNA group was decreased compared to controls. In summary, the results in our study were consisted with most of previous studies, which revealed that CD164 functioned as a tumor promoter and could promote the progression of tumors in vivo and in vitro.

CXCR4, an upstream molecule of the PI3 kinase/Akt pathway, has been shown to play critical roles in several aspects of tumor progression, such as angiogenesis, metastasis, and survival.^33^, ^34^, ^35^, ^36^, ^37^ An earlier study suggested that CD164 acted as a component of a CXCR4 complex and regulated the migration of CD133^+^ cells. 38 In recent years, many studies have indicated that CD164 played a role in tumor progression by interacting with CXCR4 and regulating the downstream of CXCR4 pathway.^23^ Knocking‐down of CD164 could up‐regulate PTEN and inhibit the activities of PI3K/AKT pathway.^13^ Besides, CD164 promoted lung tumor‐initiating cells with stem cell activity and determined tumor growth and drug resistance through Akt/mTOR signaling pathway.^14^ Therefore, we suspected that CD164 may also play roles in tumor progression of BC through CXCR4/AKT signaling pathway. More studies in the future were needed to confirm the mechanism of CD164 in BC.

In conclusion, our study was the precedent experiment which systematically researched the roles of STK32C in tumor progression of BC. From the results, we speculated that CD164 was a tumor promoter which could promote the progression of several carcinomas possibly by regulating CXCR4 relevant proteins and activating CXCR4/AKT signaling pathway. The results were needed to be verified, and exact mechanism of the functions of CD164 in BC should be researched and confirmed in the future.

The results of our study showed that CD164 was associated with the poor clinical outcomes of BC patients. Silencing of CD164 could inhibit the proliferation, migration, and invasion of tumor cells. In vivo, knocking down CD164 hindered the growth and metastasis of tumors in mice. In all, our results revealed that CD164, as a tumor promoter, played an essential role in tumor progression, which may become a potential treatment target of BC patients.

## COMPLIANCE WITH ETHICAL STANDARDS

All applicable international, national, and/or institutional guidelines for the care and use of animals were followed.

## CONFLICT OF INTEREST

None declared.
